# Supplementary material for: A mutualism without honeydew: what benefits for Melissotarsus emeryi ants and armored scale insects (Diaspididae)?
Source: PeerJ. 2017 Jul 25;5:e3599. doi: 10.7717/peerj.3599 (PMC5530990; doi:10.7717/peerj.3599)

A mutualism without honeydew: what benefits for *Melissotarsus emeryi* ants and armoured scale insects (Diaspididae)?

Christian Peeters[1*], Imré Foldi[2], Danièle Matile-Ferrero[2],
Brian L. Fisher[3]

Supplemental files

Fig. S1 Comparisons of 'yellow vacuoles' found in both diaspidids and ant guts (A) whole mount of adult diaspidid; (B) blind-ended gut of large larva; (C) midgut of ant worker (note empty crop and malpighian tubules on either extremities).

Fig. S2 White deposits around the edges of smaller female diaspidids in galleries abandoned by ants. These are likely to be wax filaments.

Fig. S3 Silk strands spun by ants during emergency repairs of galleries.

Fig. S4 Exuviae of diaspidids found in galleries abandoned by ants.

Fig. S1 Comparisons of 'yellow vacuoles' found in both diaspidids and ant guts (A) whole mount of adult diaspidid; (B) blind-ended gut of large larva; (C) midgut of ant worker (note empty crop and malpighian tubules on either extremities).

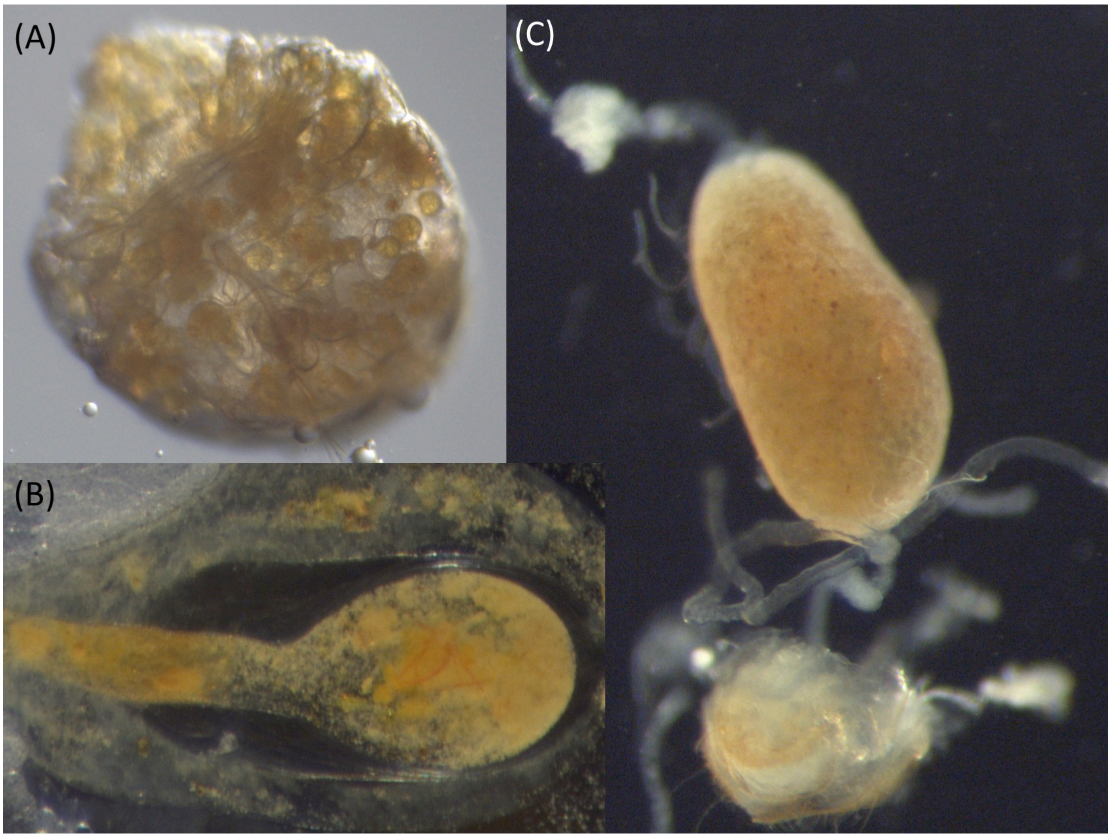

Fig. S2 White deposits around the edges of smaller female diaspidids in galleries abandoned by ants. These are likely to be wax filaments.

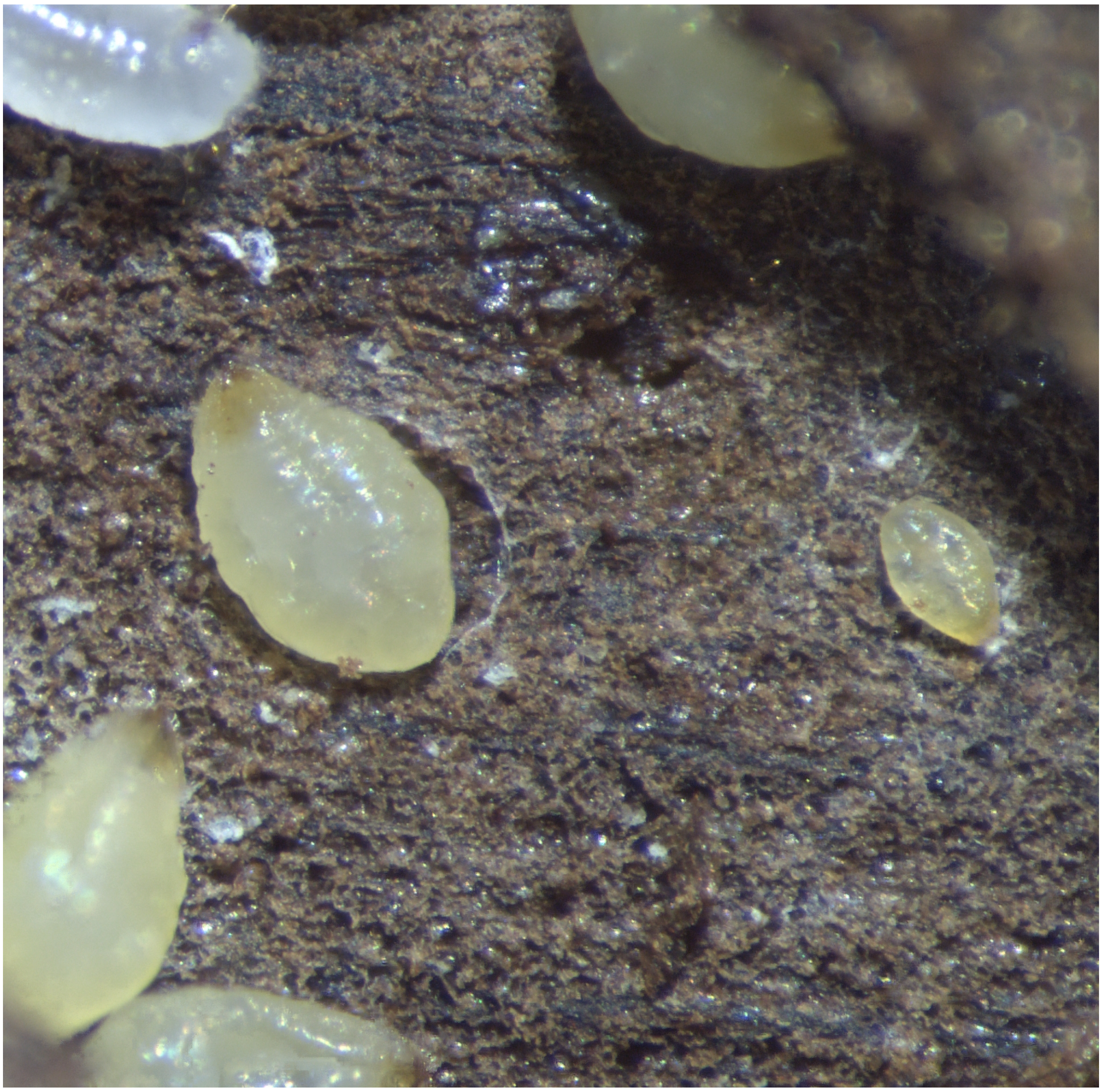

Fig. S3 Silk strands spun by ants during emergency repairs of galleries.

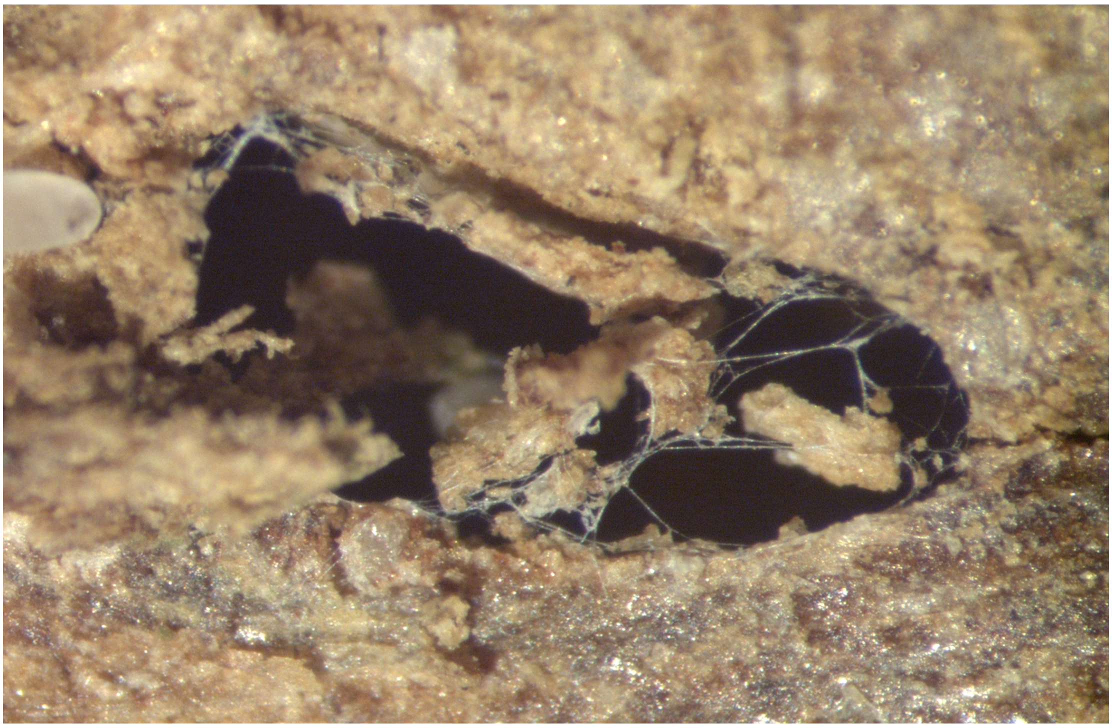

Fig. S4 Exuviae of diaspidids found in galleries abandoned by the ants.

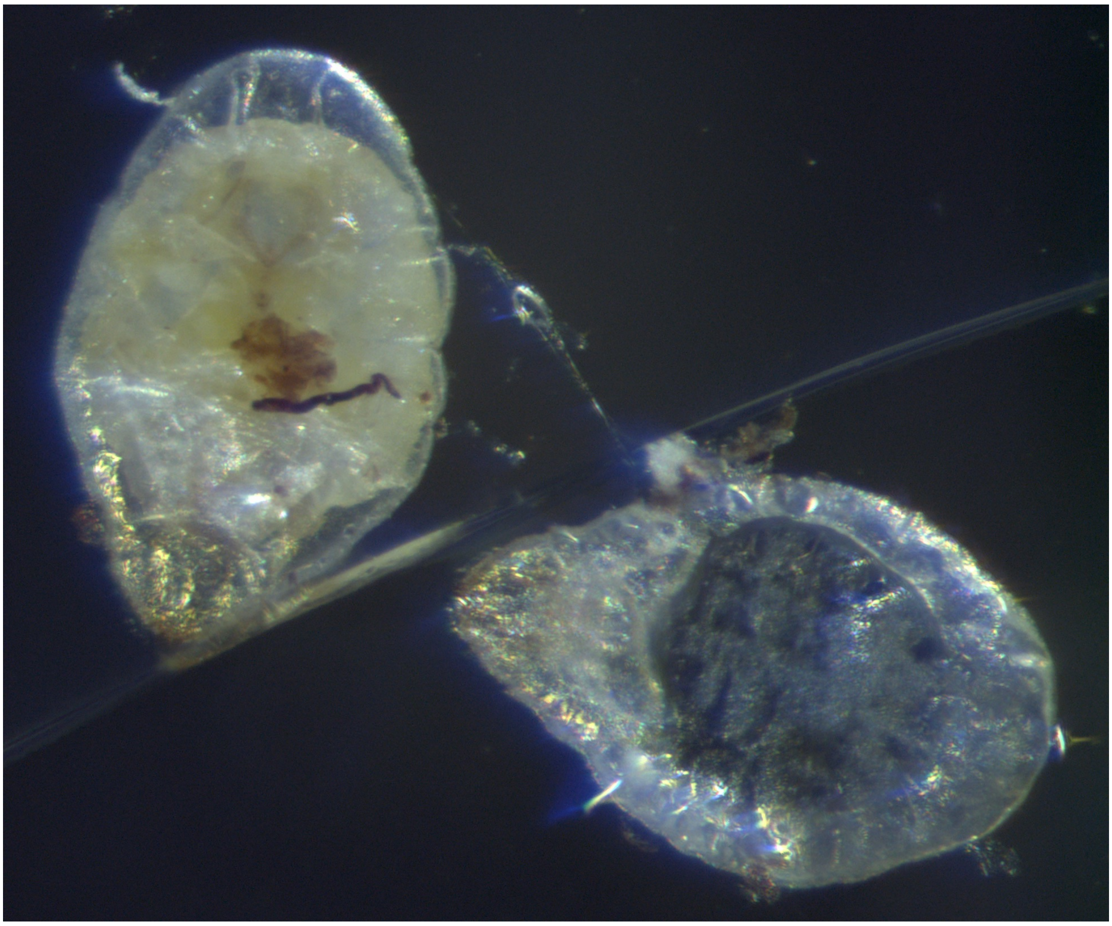

Supplement: Supplemental Information 1 [file peerj-05-3599-s001.pdf]
